# Epithelioid trophoblastic tumor coexisting with choriocarcinoma around an abdominal wall cesarean scar: a case report and review of the literature 

**DOI:** 10.1186/s13256-020-02485-8

**Published:** 2020-10-05

**Authors:** Chunfeng Yang, Jianqi Li, Yuanyuan Zhang, Hanzhen Xiong, Xiujie Sheng

**Affiliations:** 1 Department of Obstetrics and Gynecology, Third Affiliated Hospital of Guangzhou Medical University, Guangzhou, 510150 China; 2 Department of Gynecologic Oncology, National Cancer Center/Cancer Hospital, Chinese Academy of Medical Sciences & Peking Union Medical College, Panjiayuan, Chaoyang District, 100021 Beijing, People’s Republic of China; 3 Department of Pathology, Third Affiliated Hospital of Guangzhou Medical University, Guangzhou, 510150 China

**Keywords:** Mixed gestational trophoblastic tumor, Epithelioid trophoblastic tumor, Choriocarcinoma, Immunohistochemistry

## Abstract

**Background:**

Mixed gestational trophoblastic neoplasms are extremely rare and comprise a group of fetal trophoblastic tumors including choriocarcinomas, epithelioid trophoblastic tumors, and placental site trophoblastic tumors. We present a case of a patient with extrauterine mixed gestational trophoblastic neoplasm adjacent to the abdominal wall cesarean scar. On the basis of a literature review, this type of case has never been reported before due to the unique lesion location and low incidence.

**Case presentation:**

Our patient was a 39-year-old Chinese woman who had a history of two cesarean sections and one miscarriage. She had a recurrent anterior abdominal wall mass around her cesarean scar, and the mass was initially suspected of being choriocarcinoma of unknown origin. The patient had concomitant negative or mildly increased serum β-human chorionic gonadotropin at follow-up and no abnormal vaginal bleeding or abdominal pain. However, she underwent local excision twice and had two courses of chemotherapy with an etoposide and cisplatin regimen. She finally opted for exploratory laparotomy with abdominal wall lesion removal, subtotal hysterectomy, bilateral salpingectomy, and left ovarian cyst resection, which showed the abdominal wall lesion, whose components were revealed by microscopy and immunohistochemical staining to be approximately 90% epithelioid trophoblastic tumors and 10% choriocarcinomas from a solely extrauterine mixed gestational trophoblastic neoplasm around an abdominal wall cesarean scar.

**Conclusions:**

It is worth noting whether epithelioid trophoblastic tumor exists in the setting of persistent positive low-level β-human chorionic gonadotropin. More studies are required to provide mechanistic insights into these mixed gestational trophoblastic neoplasms.

## Introduction

Gestational trophoblastic neoplasms (GTNs) are a group of malignant fetal trophoblastic tumors that include choriocarcinomas (CCs), placental site trophoblastic tumors (PSTTs), and epithelioid trophoblastic tumors (ETTs). CCs are composed of variable proportions of neoplastic cytotrophoblasts, syncytiotrophoblasts, and intermediate trophoblasts. Intermediate trophoblastic lesions include exaggerated placental sites, placental site nodules (PSNs), PSTTs, and ETTs [1].

However, the exact pathogenesis of the differentiation of GTNs, especially the mixed GTNs, is still unknown because these tumors are extremely rare [2]. To our knowledge, a case of ETT coexisting with CC on an abdominal wall secondary cesarean scar has not been reported before. Here, we present our experience with the diagnosis and management of a rare case of a patient with isolated and extrauterine ETT coexisting with CC around an abdominal wall cesarean scar, in addition to a systematic review of the literature.

## Case presentation

Our patient was a 39-year-old Chinese woman who had delivered a second live full-term infant through an uncomplicated cesarean section in 2011 after a miscarriage in 2010 and a first cesarean section in 2005. In December 2014, she noticed a purple, nontender swelling appearing as an anterior abdominal wall mass around her cesarean scar. The mass was the size of a green bean and was not accompanied by any abdominal pain or abnormal vaginal bleeding. There were no notable findings in her past medical history, family history, or psychosocial history. The abdominal wall mass had progressively enlarged, which led to her presentation to a local institution. She had undergone tumor resection of the abdominal wall in June 2015, which might be interpreted as the abdominal wall endometriosis malignancy.

The patient had visited a regional tertiary hospital for a consultation regarding the pathologic diagnosis of a CC before presenting to our hospital for further diagnosis and treatment. Her physical examination showed no abnormalities except for the scar from the local excision on the abdominal wall. Our pathologists reviewed the first excised specimen from the previous hospital and confirmed the characteristic of CC coexisting with minor ETT. Her Ki-67 proliferative index was approximately 50%. Laboratory analysis revealed normal serum levels of β-human chorionic gonadotropin (β-hCG; < 1.2 IU/L) and tumor markers, including carbohydrate antigen 125 (CA 125), carcinoembryonic antigen (CEA), CA 19-9, CA 15-3, and α-fetoprotein; all of these biomarkers had consistently negative values. Positron emission tomography was performed to further determine whether other metastatic lesions existed; however, no residual tumor and suspicious malignant lesions were observed. For further evaluation, endometrial curettage was performed, the results of which revealed normal menstrual phase endometrium. Subsequently, the patient received two courses of chemotherapy with a regimen of etoposide and cisplatin (EP) over a 2-month period. During chemotherapy, her serum β-hCG levels remained negative (< 1.2 IU/L).

Subsequently, she underwent regular follow-up in the outpatient department, and a recurrent nodule was found on the same abdominal wall scar site in January 2017, approximately 17 months after the last chemotherapy. The patient was registered for admission again. Her serum β-hCG had increased to 6.17 IU/L, and two oval hypoechoic masses were visualized by ultrasonography in the subcutaneous soft tissue of the lower abdomen wall scar. Chest computed tomography (CT) and head magnetic resonance imaging (MRI) showed no abnormality. Then she underwent a second notable mass excision by ultrasound interventional localization. In this case, the nodule was in the fat layer next to the superficial fascia. Her serum β-hCG level was decreased to 3.4 IU/L on the second postoperative day. The result of pathological examination was initially in line with CC metastasis to the abdominal wall, and the patient’s Ki-67 index was 20%. However, the patient’s pathological sections were sent to the Fudan University Obstetrics and Gynecology Hospital, another tertiary hospital in China, for further consultation, and the finding was ETT. Finally, the patient was encouraged to maintain close follow-up, and her serum β-hCG level had gradually decreased.

**Fig. 1:**
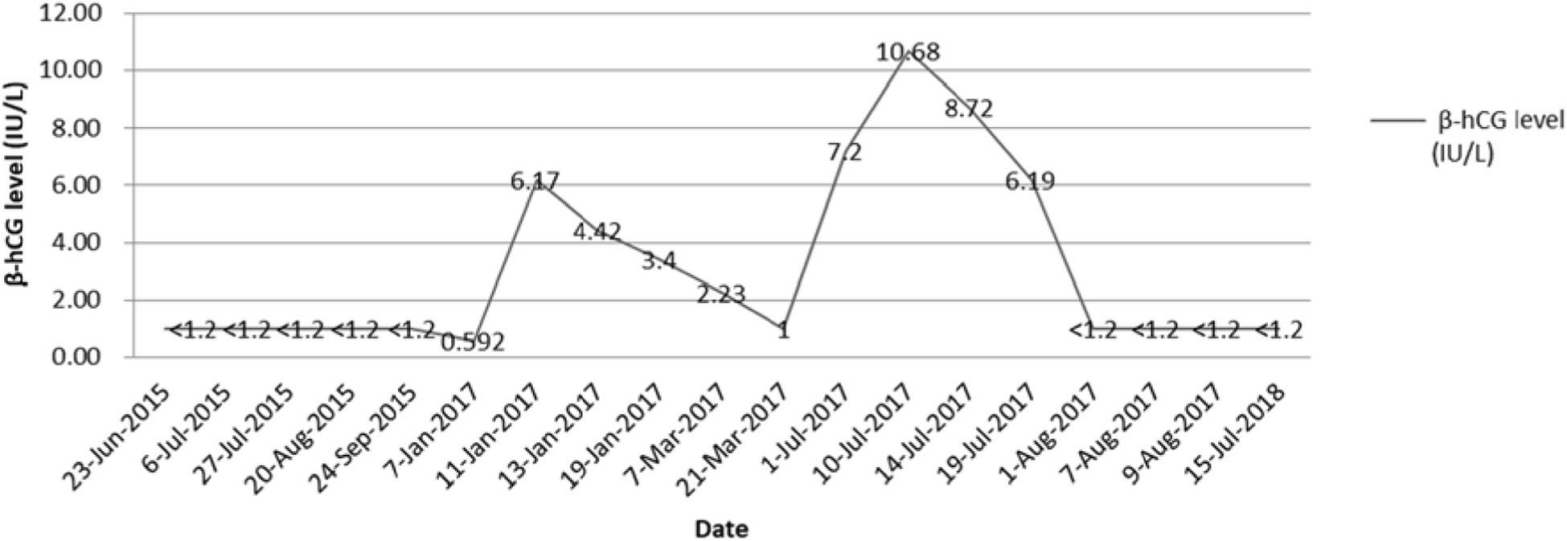
Changes of the serum β-human chorionic gonadotropin level in the patient

**Fig. 2:**
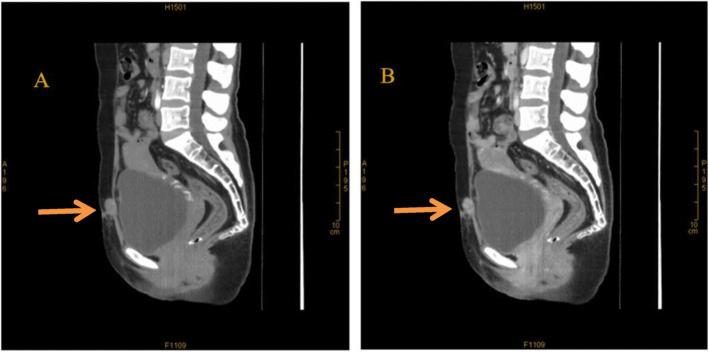
Computed tomography scan of the pelvis showing several nodules in the abdominal wall midline fascia (indicated by the arrows). The largest nodule was approximately 21 × 15 mm in size, with clear boundaries but less uniform in internal echoes. (**a**) Arterial phase enhancement, nodules mildly enhanced. (**b**) Venous phase enhancement, nodules further enhanced

In the follow-up visits, the patient’s serum β-hCG level was elevated to the highest level of 10.68 IU/L again 4 months later (Fig. 1), but she was still without any abdominal pain or abnormal vaginal bleeding. Furthermore, a pelvic CT scan showed several nodules on the abdominal wall midline fascia; the largest nodule was approximately 21 × 15 mm in size. The nodules had clear boundaries but were less uniform in internal echoes (Fig. 2a and b). Throughout the disease process, test results for tumor markers such as CA 125, CA 19-9, CEA, and HE4 were negative, and the results of routine blood sampling tests (blood cell count, liver and kidney function, coagulation function) were normal. We suggested a third resection of the mass to the patient, but she opted for a hysterectomy due to fear of malignancy and further relapse. She finally underwent exploratory laparotomy with removal of the abdominal wall lesion, subtotal hysterectomy, bilateral salpingectomy, and left ovarian cyst resection as well as right inguinal lymph node biopsy in July 2017. Intraoperative exploration revealed that the abdominal wall lesion was located on the anterior wall fascia.

**Fig. 3:**
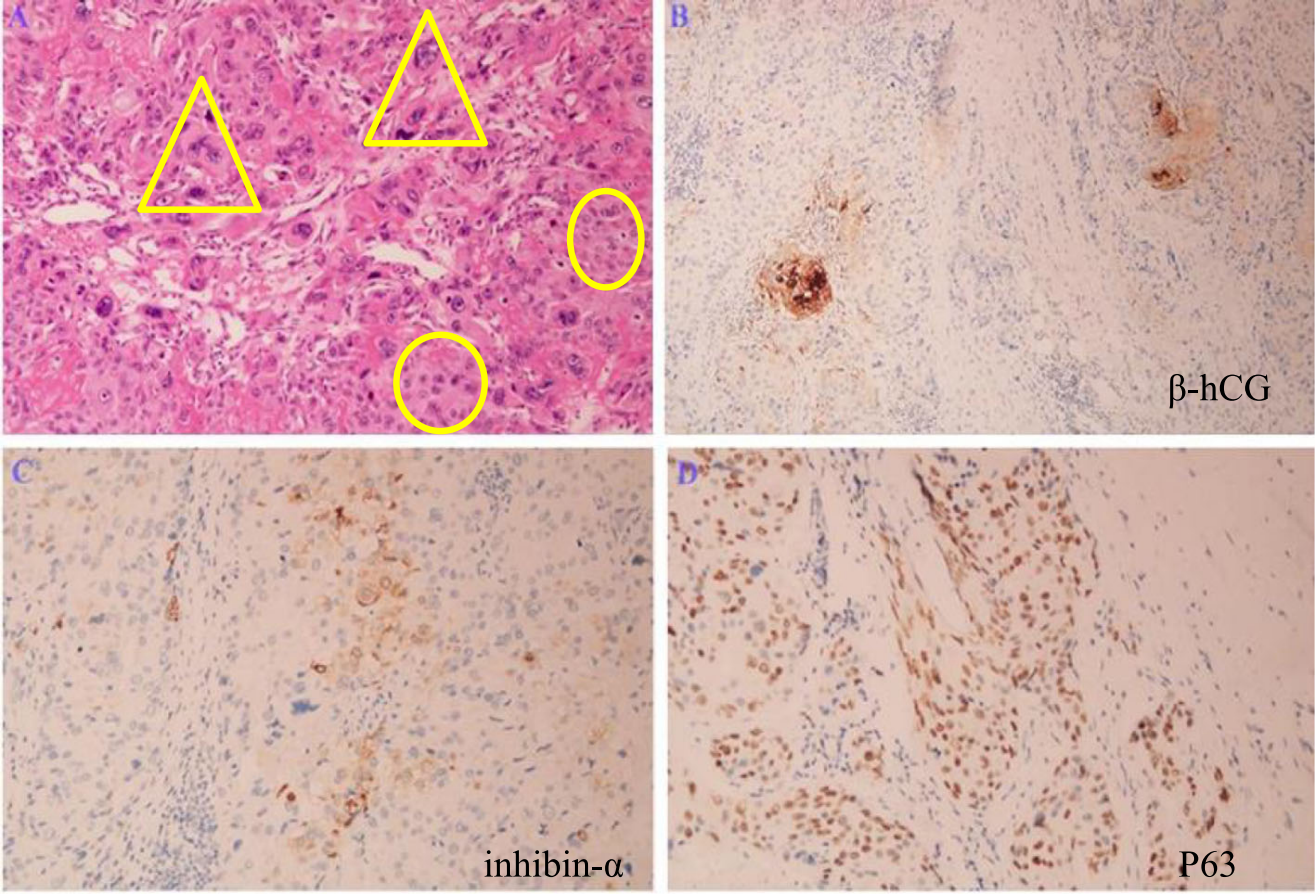
**a** The tumor was composed of nests of major epithelioid cells with necrotic debris (○) and peritumoral hyaline-like material, accompanied by scattered choriocarcinoma components (△) (Hematoxylin-Eosin (H&E) stain, original magnification × 200). **b** Focal positive cytoplasmic staining for β-human chorionic gonadotropin (original magnification × 200). **c** Cytoplasmic staining for inhibin-α was positive (+) in choriocarcinoma trophoblasts and negative (−) in epithelial trophoblasts (original magnification × 200). **d** Nuclear staining for p63 was positive (+) in both epidermoid trophoblasts and choriocarcinoma trophoblasts (original magnification × 200)

Histopathological observations suggested that hyperplasia of fatty fibrous tissue was visible with cancer infiltration and necrosis around the cancer tissue, which was consistent with trophoblastic tumor, constituted primarily by major epidermoid trophoblastic tumors (approximately 90%) and the remainder by CC components (approximately 10%) on the abdominal wall lesion (Fig. 3a). Immunohistochemistry showed β-hCG (focal positive), inhibin-α (epithelial trophoblast negative, CC positive), and p63 (epidermoid trophoblast positive, CC positive) (Fig. 3b–d). There was no tumor involvement in other tissues, including uterine, left ovarian cyst, and the right inguinal lymph nodes, which indicated an isolated and extrauterine mixed trophoblastic tumor. On the basis of all these findings, the diagnosis was ETT accompanying CC. The patient’s postoperative recovery was uneventful. Two weeks after hysterectomy, her serum β-hCG level had returned to normal (low 1.2 IU/L). Two years later, there was no evidence of recurrence according to serum β-hCG and imaging studies.

## Discussion

Mixed GTNs are extremely rare, with only 20 mixed trophoblastic tumors having been reported [2–14], including our patient’s case. According to the literature, most mixed GTNs have occurred in patients of reproductive age (age range 15–60 years) and were usually associated with a previous gestational event [2]. The interval between the preceding gestation and the diagnosis has ranged from 7 months to 38 years, and the median interval is approximately 26.2 months, which is longer for mixed GTN than for pure ETT, CC, or PSTT [2, 15]. Our patient’s age is similar to the age of onset and the intermittent period.

Mixed GTNs have clinical features more similar to those of intermediate trophoblastic tumors [2, 15]. GTN presenting in the abdominal wall is an extremely infrequent event, either as a metastasis or an isolated tumor, with only two cases of CCs and three cases of ETTs having been reported to date (Table 1) [16–19]. However, to our knowledge, this is the first report of ETT coexisting with CC as a primary and isolated extrauterine mixed trophoblastic neoplasm around an abdominal wall secondary cesarean scar, without any clinicopathologic evidence of uterine involvement. This is distinctly different from Zhang and Pan’s analysis of 20 cases of gestational trophoblastic diseases in cesarean section scars, which specified lesions located at the cesarean section scars in the uterus, not the abdominal wall [20]. The occurrence of extrauterine trophoblastic neoplasia without uterine lesion lacks sufficient explanatory information. Possible etiologies may be as follows: (1) metastatic involvement from an antecedent primary uterine tumor, which may have undergone regression after metastasis; (2) neoplastic transformation of trophoblastic cells that have spread outside the uterus during previous intrauterine pregnancy; or (3) trophoblastic differentiation from germ cell tumor or somatic cancer [21]. The origin of the mixed trophoblastic neoplasm in our patient’s case was similar to the findings for the two previously reported cases of abdominal wall ETTs around a cesarean scar, which might favor the theory of trophoblastic cell seeding due to surgery rather than a true tumor metastasis [18, 19], but we also could not rule out a metastatic lesion from a uterine tumor that had disappeared, because these trophoblastic remnants might undergo malignant change after a period of latency, although the trigger of this change is unknown [22]. We did not adopt effective microsatellite genotyping to differentiate gestational from nongestational β-hCG-producing tumors, as demonstrated by Fisher *et al.* [23], because our conditions were limiting.

**Table 1:** Summary of cases of trophoblastic neoplasms presenting on the abdominal wall

Author (year)	Age (years)	Antecedent pregnancy	Presentation and tumor size (mm)	β-hCG level (IU/L)	Extrauterine trophoblastic tumor	Surgical treatment	Pathological diagnosis	Chemotherapy	Response to chemotherapy	Follow-up (months)	Outcome
Bailey *et al.*, [16] (2003)	Young	FTVD, 3 years	Vaginal spotting, left lower anterior pelvis abdominal wall, 20 × 20	317,735	YES	Laparoscopic local resection	CC	EMA-CO	Complete response	24	NED
Mukherjee *et al.*, [17] (2013)	41	FTVD, 13 years	Anterior abdominal wall and iliopsoas mass, 77 × 51 × 34	87,643	NO	Cytoreductive laparotomy (PLND, omentectomy, and peritoneal biopsies)	CC	EMA-CO	Complete response	18	NED
Davis *et al.*, [18] (2015)	49	FTCS, 16 years	Umbilical hernia, multiple calcified nodules in the peritoneal cavity, largest diameter 85	6.9	YES.	TAH-BSO, omentectomy, right ureterolysis, and extensive resection of the pelvic peritoneum	ETT	EMA-EP prior to surgery	No response	18	Stable disease: residual small bowel mesentery
Davis *et al.*, [18] (2015)	44	FTCS, 22 years	Pain at a prior cesarean section scar, diameter 90	Unknown	YES	Diagnostic laparoscopy, resection of abdominal wall mass	ETT	EMA-EP	Partial response	26	Progressive disease: pubic symphysis metastasis
Hsiue *et al.*, [19] (2017)	54	FTSCS, 23 years	Anterior abdominal cesarean scar mass, 57 × 32 enlarged to diameter 76 Postoperative recurrence: diameter 46 and multiple intestinal metastases	8.36	YES	1. Incisional biopsy 2. Wide excision 3. Resected recurrent left lower abdominal wall tumor and multiple intestinal metastases	ETT	1. Prior to surgery: EMA-EP 2. After the second surgery: ifosfamide, etoposide	1. No response 2. Under follow-up	2	2.6-cm size of peritoneal tumor at anterior pelvic cavity
Our patient	39	FTSCS, 3 years	Anterior abdominal wall cesarean scar mass, fingertip size	< 1.2	YES	1. Wide local excision, twice 2. TAH-BSO	90% ETT + 10% CC	Prior to the first surgery: EP	No response	12	NED

*Abbreviations: EMA-CO* Etoposide, methotrexate, dactinomycin, cyclophosphamide, vincristine, *EMA-EP* Etoposide, methotrexate, actinomycin-D, cisplatin, *FTCS* Full-term cesarean section, *FTSCS* Full-term second cesarean section, *FTVD* Full-term vaginal delivery, *NED* No evidence of disease, *PLND* Pelvic lymph node dissection, *RA* Robot-assisted, *TAH-BSO* Total abdominal hysterectomy, bilateral salpingo-oophorectomy

The distinction of ETT from other trophoblastic tumors is more challenging. Unlike CCs and hydatidiform moles, the serum β-hCG level with ETT is usually within the normal range or is slightly elevated, as Palmer *et al*. reported [22]. Our patient’s serum β-hCG level was negative or showed a slight elevation at follow-up, which made the diagnosis more difficult. Accurate interpretation of MRI, CT, and sonographic image findings might be helpful in differentiating ETT from other gestational trophoblastic diseases and carcinomas; however, imaging specialists were not so familiar with them, which made it was difficult to identify them because of their rarity [24]. Pathologically, typical ETTs are composed of a relatively uniform population of mononuclear intermediate trophoblastic cells with eosinophilic cytoplasm surrounded by a well-defined cell membrane, whereas CCs contain the dimorphic trophoblastic population of cells [3]. Immunostaining for β-hCG is only focally positive in ETT but is diffusely positive in CC. In addition, the Ki-67 proliferative index helps in the differential diagnosis, as it is very high (> 50%) in CC and squamous cell carcinoma but relatively lower in PSTT (15–25%), ETT (10–25%), and PSN (< 10%) [3]. More immunohistochemical panels, including human placental lactogen, inhibin-α, and p63, are helpful in establishing a correct diagnosis [3, 25]. However, it is more complicated to analyze coexisting trophoblastic tumors. Several factors made diagnosis in our patient’s case quite challenging. One was that the tumor occurred at a rare and special site on the abdominal wall without evidence of disease in the uterus. Another factor was our patient’s serum β-hCG level, which remained negative when the first diagnosis was made and only exhibited a mildly incidental increase at follow-up.

The pathogenesis of mixed trophoblastic tumors is not well known. According to the model presented by Mao *et al*., CC was the most primitive trophoblastic tumor, whereas PSTT or ETT was relatively more differentiated [25]. Furthermore, the proportion of different trophoblastic subpopulations differentiating toward intermediate trophoblasts or syncytiotrophoblasts in a given specimen is variable, depending on the tumor microenvironment. This hypothesis explains the existence of gestational trophoblastic neoplasia with mixed histological features, including CC, PSTT, and ETT. In these cases, it was plausible that chemotherapeutic agents eradicated the more primitive cytotrophoblastic components, permitting differentiation of chorionic-type intermediate trophoblastic cells, which were more refractory to chemotherapy than the original CC components [25]. This is also the reason why ETT predominates over CC after chemotherapy. One research group proposed “postchemotherapy GTN,” which shared overlapping features with ETT, even though it had an indolent behavior that differed from that of ETT, which showed an aggressive clinical course and adverse prognosis [26]. However, postchemotherapy GTN could overgrow and eventually develop into ETT once the cytotoxic effect of chemotherapy was eliminated, similar to a “snapshot” of the CC–ETT transition [26]. These findings are further supported the speculation of that ETT developed from a preexisting CC after chemotherapy [27].

After our patient presented to the hospital, she received two chemotherapy regimens with EP after the first local resection of the abdominal wall mass. Our pathologists had made a mistake in interpreting the second surgical specimen; other pathologists from different tertiary hospitals realized it was an ETT, not a CC, upon analyzing the same specimen. With the third recurrence, the final pathologic diagnosis was predominantly ETT coexisting with a small proportion of CC that was only present on the abdominal wall lesion. On the basis of the above information, we could not rule out the possibility that the predominant ETT lesion might be associated with the previous CC. However, there is still not enough evidence to support the notion that the predominant ETT was a result of previous chemotherapy.

Currently, there is extremely limited experience with ETT coexisting with CC. CC is sensitive to chemotherapeutics, but PSTT and ETT are not. Total hysterectomy with lymph node dissection was recommended for mixed GTNs, and chemotherapy should be used in patients with metastatic disease or in patients without metastatic disease but with adverse prognostic factors [2]. Furthermore, peripheral stem cell support might have potential for use in future patients with relapse of CC [8]. The clinicopathologic presentations of mixed trophoblastic tumors are unclear. Whether their behaviors depend on the proportion of different components needs to be further studied, and the optimal multimodal treatment approach needs to be determined.

## Conclusions

Mixed gestational trophoblastic tumors are rare. We report the first case of extrauterine mixed trophoblastic tumor around an abdominal wall cesarean scar. It is essential to distinguish ETT from PSTT, CC, and other malignancies, especially in normal tissue in the uterus. Pathologists and clinicians should pay more attention to mutual communication and arrive at a correct diagnosis and finally select an optimal therapeutic schedule. Because limited data are available related to mixed GTNs, long follow-up is necessary, and additional cases must be accumulated.

## Data Availability

Clinical data and the accompanying examinations are available according to the authorization from the patient.
